# Supplementary material for: The slit diaphragm in Drosophila exhibits a bilayered, fishnet architecture
Source: Nat Commun. 2025 Oct 1;16:8741. doi: 10.1038/s41467-025-64347-5 (PMC12489029; doi:10.1038/s41467-025-64347-5)
Supplement: Supplementary file 1 — Supplementary Information [file 41467_2025_64347_MOESM1_ESM.pdf]

Table of contents - Supplementary Information

Supplementary Figure 1: RT-TEM images and Airyscan LM illustrate the labyrinthine channels forming the nephrocyte filtration barrier.

Supplementary Figure 2: The wild-type nephrocyte slit diaphragm seen from various perspectives in cryo-electron tomograms.

Supplementary Figure 3: Comparison of the resolution in nephrocytes between conventional room temperature transmission electron microscopy (RT-TEM) and cryo-electron tomography (cryo-ET) in wild-type animals.

Supplementary Figure 4: Reduction of slit diaphragm length following temperature-induced activation of *sns*-RNAi.

Supplementary Figure 5: Confirmation of effective *sns* silencing by immunofluorescence.

Supplementary Table 1: Additional fly strains.

Supplementary Table 2: Cryo-ET data collection parameters.

Supplementary Table 3: Cryo-ET processing parameters for subtomogram averaging.

Supplementary Table 4: Primary antibodies.

Supplementary References.

Supplementary Figures

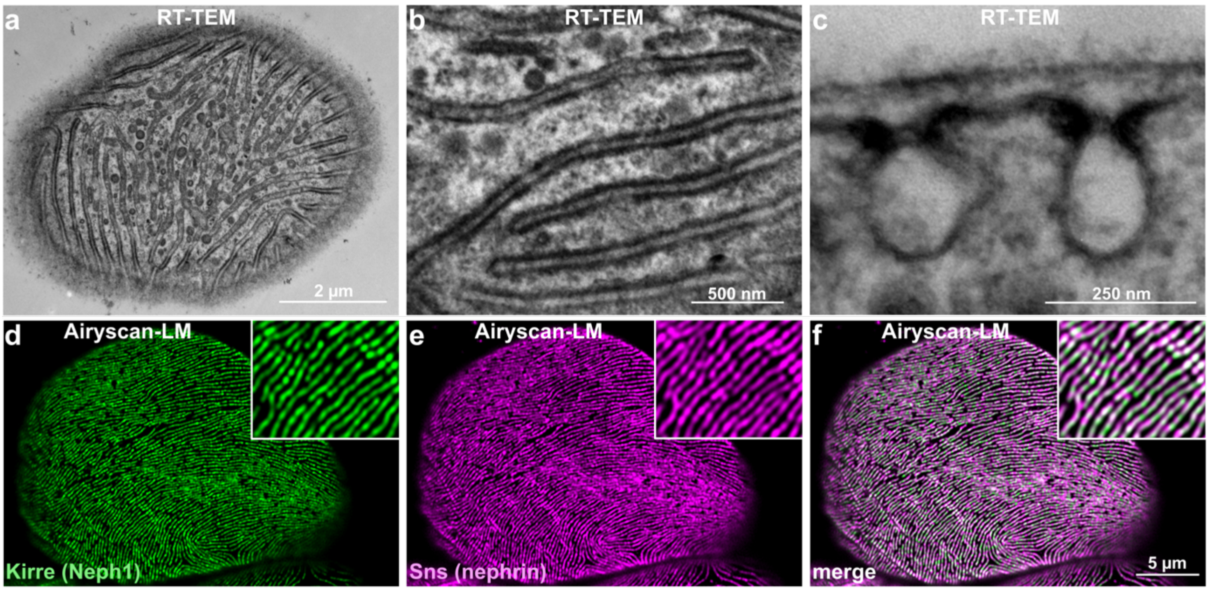

Supplementary Figure 1: RT-TEM images and Airyscan LM illustrate the labyrinthine channels forming the nephrocyte filtration barrier.

(a-c) RT-TEM images of control nephrocytes are shown. **(a-b)** Tangential sections illustrate the fingerprint-like pattern formed by parallel lines of slit diaphragms spanning the labyrinthine channels, **(a)** which are discernible by parallel electron-dense lines with higher magnification **(b)**. **(c)** Magnified RT-TEM image of a nephrocyte surface detail in a cross-section shows slit diaphragms as thin lines between electron-dense areas on top of the oval membrane invagination. **(d-f)** Immunofluorescence microscopy image acquired in Airyscan mode shows the colocalization of slit diaphragm proteins Sns (ortholog of nephrin) and Kirre (ortholog of Neph1) in a fingerprint-like pattern.

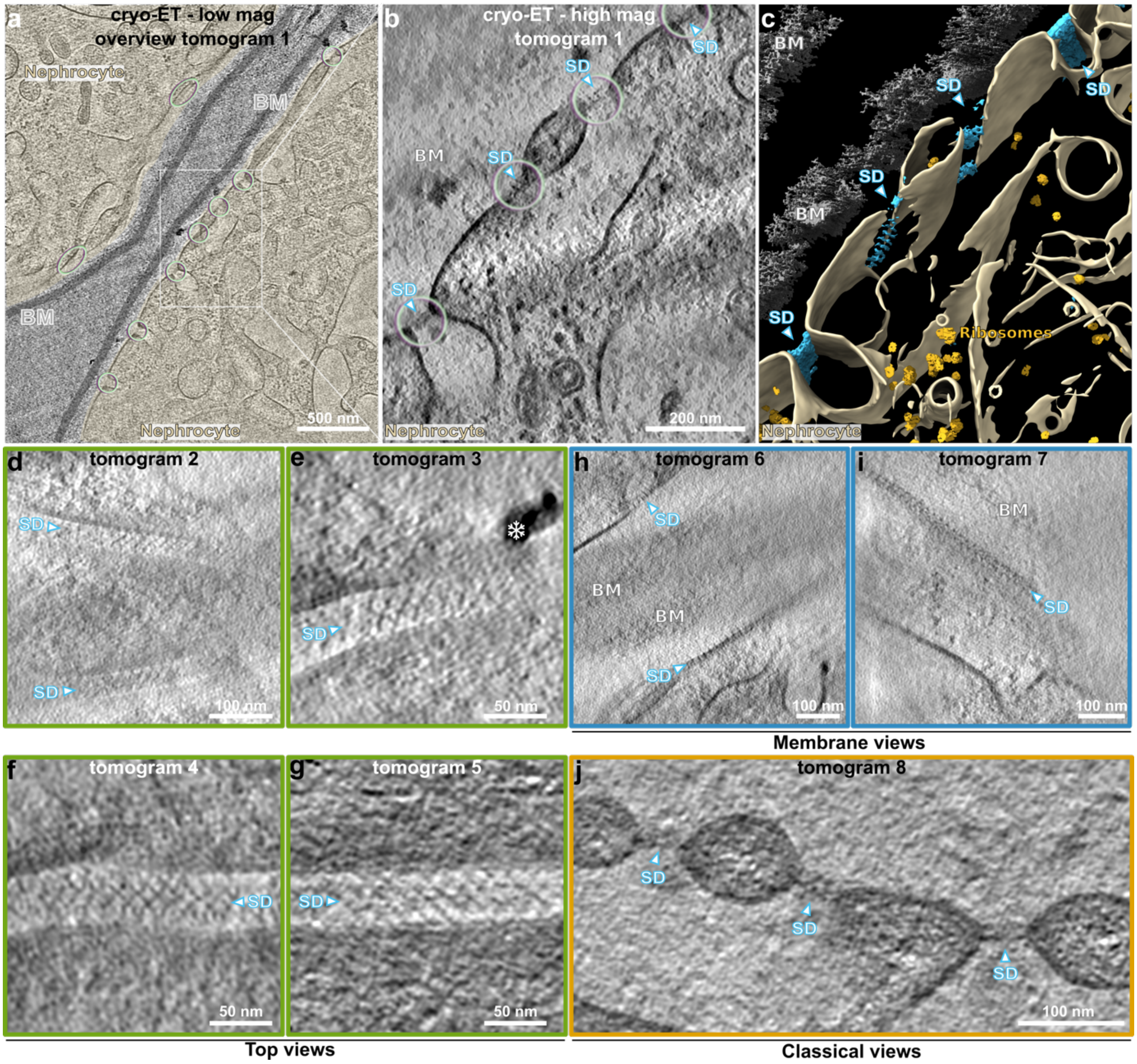

Supplementary Figure 2: The wild-type nephrocyte slit diaphragm (SD) seen from various perspectives in cryo-electron tomograms.

(a) Slice of a low magnification tomographic reconstruction showing the crosssection of two wild-type nephrocytes (highlighted in light yellow), with invaginations spanned by the slit diaphragm (indicated by circles). The basement membrane (BM) is highlighted in gray. **(b)** Slice of a high magnification tomogram acquired at the same region of interest, revealing the classical view of the SD and an oblique view (indicated by arrows and circles). All slices of the full tomogram are shown in Supplementary Movie 3. **(c)** Segmentation of the tomogram in (b), displaying the 3D architecture of the nephrocyte and the SD bridging the labyrinthine channels. Beige: membranes, blue: nephrocyte SDs, gray: basement membrane (BM), bright yellow: ribosomes**. (d-g)** Slices of 4 different high magnification tomographic reconstructions, revealing top views of the SD, indicated by arrows. Snowflake: ice contamination. **(h-i)**

Slices of 2 different high magnification tomographic reconstructions, revealing membrane views of the SD. BM: basement membrane, **(j)** Slice of a high magnification tomographic reconstruction revealing classical views of the SD. The data for the eight tomograms shown in this figure was acquired on three lamellae from three different wild-type *Drosophila* nephrocyte samples. The full tomographic reconstructions for (b) and (f) are shown in Supplementary Movies 3 and 4, respectively.

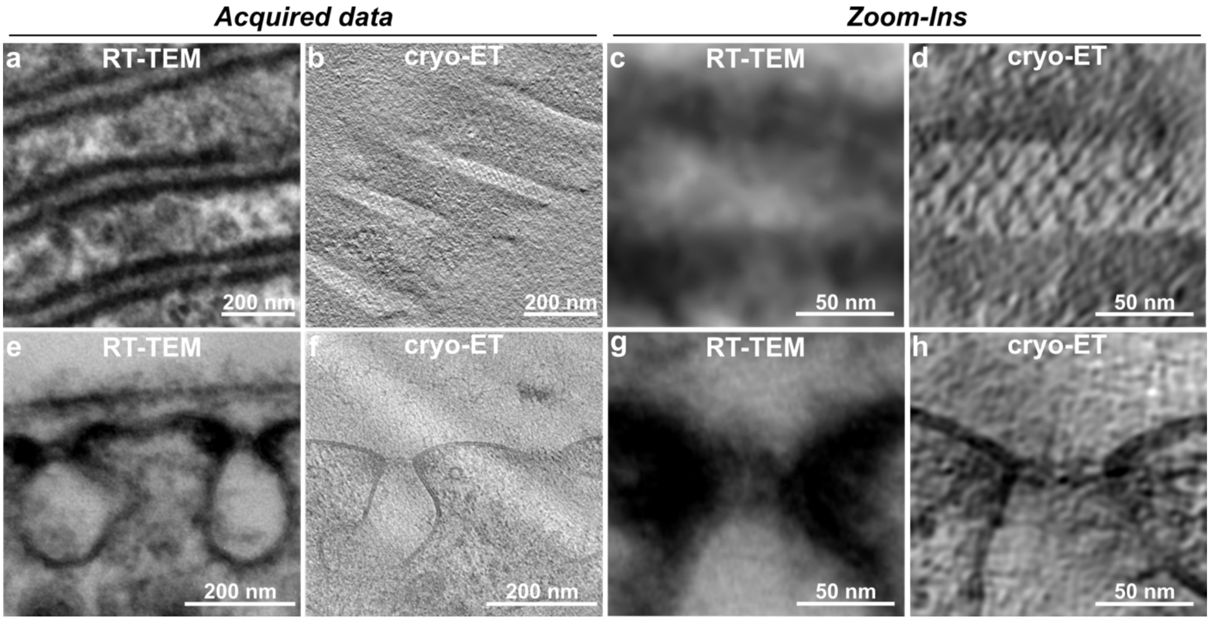

Supplementary Figure 3: Comparison of the image quality and the resolution in nephrocytes between conventional room temperature transmission electron microscopy (RT-TEM) and cryo-electron tomography (cryo-ET) in wild-type animals.

(a, e) RT-TEM overview images of slit diaphragms (SDs) shown in tangential and in the cross-section, respectively. **(b, f)** Slices of the tomographic reconstructions obtained by cryo-ET for the tangential section and cross-section across SDs, at the same magnification as the RT-TEM images shown for comparison. The bi-layered (f), fishnet-like (b) SD can be seen in the extracellular space within the channels. A richer image can be appreciated, displaying individual ribosomes, cytoskeletal fibers, and many cellular features that are absent in the RT-TEM images. **(c, g)** Zoom-ins of RT-TEM images of the SD shown in tangential (c) and in the cross-section (g). **(d, h)** Zoom-ins of the tomographic reconstructions of the cryo-ET dataset, revealing the fishnet-like arrangement (d) and the bi-layered architecture (h) of the SD, which is not discernible in the resolution that is possible using RT-TEM.

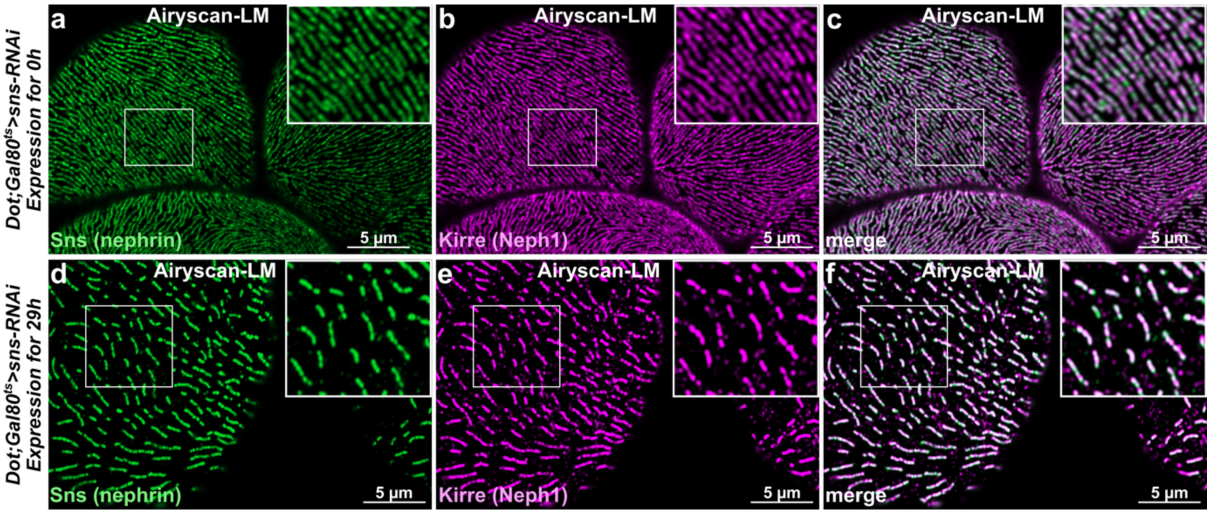

Supplementary Figure 4: Reduction of slit diaphragm length following temperature-induced activation of *sns*-RNAi.

(a-f) Fluorescence microscopy images of nephrocytes acquired in Airyscan mode. Nephrocytes before temperature-sensitive silencing of *sns* show a wildtype fingerprint pattern with long, parallel slit diaphragm lines **(a-c).** After acute silencing of *sns* for 29 hours, the slit diaphragm lines are noticeably shortened (**d-f**, images are taken from **Fig. 4 a-c** for comparative purposes).

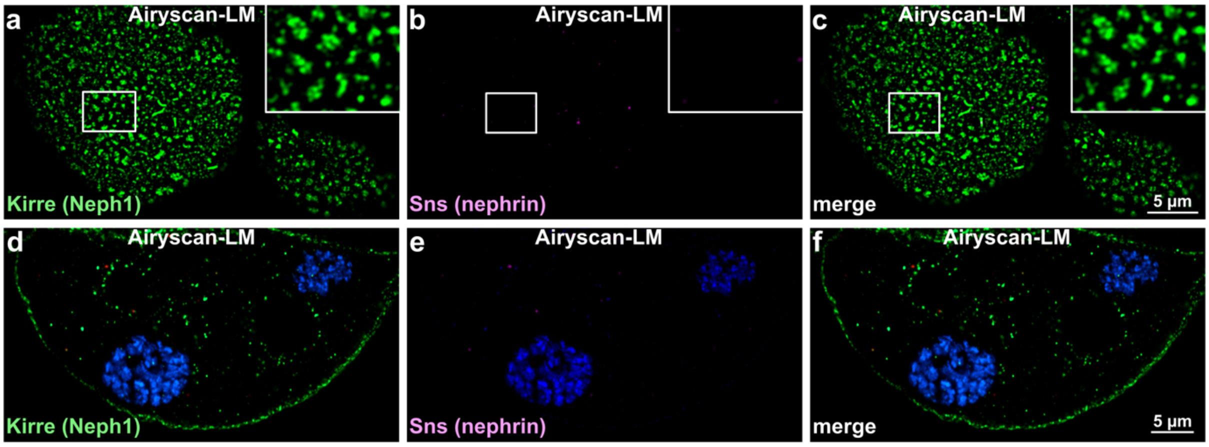

Supplementary Figure 5: Confirmation of effective *sns* silencing by immunofluorescence.

(a-f) Fluorescence microscopy images acquired in Airyscan mode of nephrocytes after prolonged silencing of *sns* in tangential (a-c) and cross-section (d-f) illustrate the absence of Sns (nephrin) protein. The slit diaphragm protein Kirre (Neph1) exhibits round clusters (a) instead of the normal linear pattern without its binding partner.

Supplementary Tables

Supplementary Table 1: All fly strains

**Fly strain**	**Source**
tubP-GAL80^{ts}	Bloomington Drosophila Stock Center #7019
UAS-EGFP-RNAi	Bloomington Drosophila Stock Center #41553
Dorothy-GAL4	Bloomington Drosophila Stock Center #6903
UAS-*Rab5*-RNAi	Bloomington Drosophila Stock Center #34832
Myc-Sns	Lang et al., 2022; PMID: 35876643[1]
UAS-*sns*-RNAi	Vienna Drosophila RNAi Center #109442
UAS-*sns*-RNAi	Bloomington Drosophila Stock Center #64872
Prospero-GAL4	Weavers et. al., 2009; PMID:18971929[2]

Supplementary Table 2: Cryo-ET data collection parameters.

**Microscope**	FEI Titan Krios G2
**Detector**	Gatan K3
**Acquisition Software**	SerialEM v.4.1 beta[3]
**Magnification**	33 kx
**Voltage [kV]**	300
**Electron exposure per micrograph [e^-/Å^2]**	2.5 to 3.125
**Defocus range [µm]**	-3 to -6.5
**Number of frames**	17
**Images per tiltseries**	41 to 52
**Increment step**	2° to 3°
**Pixel size [Å] (super-resolution)**	1.34

Supplementary Table 3: Cryo-ET processing parameters for subtomogram averaging.

**Tilt series acquired**	45
**Tilt series used**	16
**Initial particle number**	848
**Final particle number**	595
**Map resolution [Å]**	39.58
**FSC threshold**	0.143
**Symmetry imposed**	C1
**Motion correction**	MotionCor2[4] (RELION-5[5] implementation)
**CTF estimation**	CTFFIND-4.1.14[6]
**Tilt series alignment**	IMOD (v.4.11.24) patch tracking[7]<br>or AreTomo2[8]
**Tomographic reconstruction**	RELION-5.0[5]
**Subtomogram averaging**	RELION-5.0[5]

Supplementary Table 4: Primary antibodies

Antibody	Source
mouse anti-Myc	Cell Signaling Technologies #2276<br><br>Dilution: 1:200
rabbit anti-Kirre	Wolff et al, 2025; PMID: 40316169[9]<br><br>Dilution: 1:500
guinea pig anti-Sns	Milosavljevic et. al, 2022; PMID: 36137753[10]<br><br>Dilution: 1:100

SUPPL. REFERENCES

1 Lang, K. *et al.* Selective endocytosis controls slit diaphragm maintenance and dynamics in Drosophila nephrocytes. *Elife* **11** (2022). https://doi.org/10.7554/eLife.79037

2 Weavers, H. *et al.* The insect nephrocyte is a podocyte-like cell with a filtration slit diaphragm. *Nature* **457**, 322-326 (2009). https://doi.org/10.1038/nature07526

3 Mastronarde, D. N. SerialEM: A Program for Automated Tilt Series Acquisition on Tecnai Microscopes Using Prediction of Specimen Position. *Microscopy and Microanalysis* **9**, 1182-1183 (2003). https://doi.org/10.1017/s1431927603445911

4 Zheng, S. Q. *et al.* MotionCor2: anisotropic correction of beam-induced motion for improved cryo-electron microscopy. *Nat Methods* **14**, 331-332 (2017). https://doi.org/10.1038/nmeth.4193

5 Burt, A. *et al.* An image processing pipeline for electron cryo-tomography in RELION-5. *FEBS Open Bio* **14**, 1788-1804 (2024). https://doi.org/10.1002/2211-5463.13873

6 Rohou, A. & Grigorieff, N. CTFFIND4: Fast and accurate defocus estimation from electron micrographs. *J Struct Biol* **192**, 216-221 (2015). https://doi.org/10.1016/j.jsb.2015.08.008

7 Mastronarde, D. N. & Held, S. R. Automated tilt series alignment and tomographic reconstruction in IMOD. *J Struct Biol* **197**, 102-113 (2017). https://doi.org/10.1016/j.jsb.2016.07.011

8 Zheng, S. *et al.* AreTomo: An integrated software package for automated marker-free, motion-corrected cryo-electron tomographic alignment and reconstruction. *J Struct Biol X* **6**, 100068 (2022). https://doi.org/10.1016/j.yjsbx.2022.100068

9 Wolff, J. M. *et al.* Transgenic human nephrin in Drosophila nephrocytes facilitates variant analysis. *Kidney Int* **108**, 57-73 (2025). https://doi.org/10.1016/j.kint.2025.03.030

10 Milosavljevic, J. *et al.* Nephrotic Syndrome Gene TBC1D8B Is Required for Endosomal Maturation and Nephrin Endocytosis in Drosophila. *J Am Soc Nephrol* **33**, 2174-2193 (2022). https://doi.org/10.1681/ASN.2022030275
