# Supplementary material for: The slit diaphragm in Drosophila exhibits a bilayered, fishnet architecture
Source: Nat Commun. 2025 Oct 1;16:8741. doi: 10.1038/s41467-025-64347-5 (PMC12489029; doi:10.1038/s41467-025-64347-5)
Supplement: Supplementary file 2 — Description of Additional Supplementary File [file 41467_2025_64347_MOESM2_ESM.pdf]

The Description of Additional Supplementary Files

Supplementary Movie 1: Three-dimensional visualization of Sns localizing to the labyrinthine channels of nephrocytes. Visualization with IMARIS software highlights the linear and dot-like patterns formed by the labyrinthine channels, visible in both tangential and cross-sectional views of the nephrocytes. The three-dimensional reconstruction is based on Airyscan fluorescence microscopy of nephrocytes stained for Myc-Sns.

Supplementary Movie 2: Cryo-ET of a wild-type Drosophila nephrocyte revealing top views of the slit diaphragm (SD) Three dimensional reconstruction of a cryo-ET dataset revealing the wild-type SD from various perspectives. Especially the top view becomes apparent, clearly showing the fishnet architecture of the SD. The cryo-ET density map, obtained by subtomogram averaging, is shown overlaid with the tomographic data. Scale bar: 200nm.

Supplementary Movie 3: Cryo-ET of a wild-type Drosophila nephrocyte revealing classical and membrane views of the slit diaphragm (SD) Three dimensional reconstruction of cryo-ET data from a wild-type nephrocyte, revealing both classical views and membrane views of the slit diaphragm. These views highlight the bilayered architecture and the periodicity of the fishnet-like SD. Scale bar: 200nm.

Supplementary Movie 4: Cryo-ET of a wild-type Drosophila nephrocyte revealing top and membrane views of the slit diaphragm (SD) Three dimensional reconstruction of cryo-ET data from a wild-type nephrocyte. Both membrane views and top views of the SD can be observed, displaying the fishnet architecture of the SD. Scale bar: 200nm.
