# Supplementary material for: The slit diaphragm in Drosophila exhibits a bilayered, fishnet architecture
Source: Nat Commun. 2025 Oct 1;16:8741. doi: 10.1038/s41467-025-64347-5 (PMC12489029; doi:10.1038/s41467-025-64347-5)
Supplement: Supplementary file 7 — Reporting Summary [file 41467_2025_64347_MOESM7_ESM.pdf]

nature portfolio

Corresponding author(s): Achilleas S. Frangakis

Last updated by author(s): Aug 28, 2025

Reporting Summary

Nature Portfolio wishes to improve the reproducibility of the work that we publish. This form provides structure for consistency and transparency in reporting. For further information on Nature Portfolio policies, see our Editorial Policies and the Editorial Policy Checklist.

Statistics

For all statistical analyses, confirm that the following items are present in the figure legend, table legend, main text, or Methods section.

n/a	Confirmed	
☐	☒	The exact sample size (*n*) for each experimental group/condition, given as a discrete number and unit of measurement
☐	☒	A statement on whether measurements were taken from distinct samples or whether the same sample was measured repeatedly
☒	☐	The statistical test(s) used AND whether they are one- or two-sided<br>*Only common tests should be described solely by name; describe more complex techniques in the Methods section.*
☒	☐	A description of all covariates tested
☒	☐	A description of any assumptions or corrections, such as tests of normality and adjustment for multiple comparisons
☒	☐	A full description of the statistical parameters including central tendency (e.g. means) or other basic estimates (e.g. regression coefficient) AND variation (e.g. standard deviation) or associated estimates of uncertainty (e.g. confidence intervals)
☒	☐	For null hypothesis testing, the test statistic (e.g. F, t, r) with confidence intervals, effect sizes, degrees of freedom and P value noted<br>*Give P values as exact values whenever suitable.*
☒	☐	For Bayesian analysis, information on the choice of priors and Markov chain Monte Carlo settings
☒	☐	For hierarchical and complex designs, identification of the appropriate level for tests and full reporting of outcomes
☒	☐	Estimates of effect sizes (e.g. Cohen's d, Pearson's r), indicating how they were calculated

Our web collection on statistics for biologists contains articles on many of the points above.

Software and code

Policy information about availability of computer code

Data collection	open source:<br>SerialEM v 4.1 beta (doi:10.1017/s1431927603445911);<br>closed source:<br>ZEN 2009 blue edition v 2.1 (Carl ZEISS)<br>STEDYCON smart control
Data analysis	Freeware and commercial code has been used as referenced in the manuscript.<br>open source:<br>IMOD v 4.11.24 (doi:10.1016/j.jsb.2016.07.011);<br>Fiji/ImageJ v 2.9.0/1.53t (doi:10.1038/nmeth.2019, doi:10.1002/mrd.22489);<br>Motioncor2 (doi:10.1038/nmeth.4193);<br>CTFFIND4 v4.1.13 (doi:10.1016/j.jsb.2015.08.008);<br>tom_deconv.m from TOM toolbox (https://github.com/dtegunov/tom_deconv, doi:10.1016/j.jsb.2004.10.006);<br>cryoCARE v 0.1.1 (doi.:10.1016/bs.mcb.2019.05.001);<br>IsoNet v 0.2 (doi:10.1038/s41467-022-33957-8)<br>AreTomo2 (https://github.com/czimaginginstitute/AreTomo2)<br>EmSART, part of the Artiatomi software package (https://github.com/uermel/Artiatomi);<br>UCSF ChimeraX v 1.8 (doi:10.1002/pro.4792);<br>ArtiaX v 0.5 (doi: 10.1002/pro.4472);<br>mcm-cryoet (https://github.com/FrangakisLab/mcm-cryoet, doi: 10.1016/j.jsb.2022.107833);<br>RELION-5.0 (http://www.github.com/3dem/relion, doi:10.1002/2211-5463.13873);

Coot v 0.9.8 (doi:10.1107/S0907444910007493)
AlphaFold3 (doi: 10.1038/s41586-024-07487-w)
GIMP 3.0.2-1 (https://www.gimp.org)

closed source:
MATLAB 2022b (https://www.mathworks.com);
Dragonfly Version 2024.1 for Windows (https://www.theobjects.com/dragonfly);
Imaris 10.2 (https://imaris.oxinst.com/)
SVI Huygens (Scientific Volume Imaging, The Netherlands, http://svi.nl)

For manuscripts utilizing custom algorithms or software that are central to the research but not yet described in published literature, software must be made available to editors and reviewers. We strongly encourage code deposition in a community repository (e.g. GitHub). See the Nature Portfolio guidelines for submitting code & software for further information.

Data

Policy information about availability of data

All manuscripts must include a data availability statement. This statement should provide the following information, where applicable:

- Accession codes, unique identifiers, or web links for publicly available datasets
- A description of any restrictions on data availability
- For clinical datasets or third party data, please ensure that the statement adheres to our policy

No large-scale data sets from high-throughput analyses were generated or analyzed in this study. The cryo-ET structure solved in this study is available in the Electron Microscopy Data Bank (EMDB) under the accession code EMD-53557 [https://www.ebi.ac.uk/emdb/EMD-53557]. Atomic coordinates of the previously determined X-ray structure used in this study is available in the Protein Data Bank (PDB) under the following accession code: 4OFY [https://doi.org/10.2210/pdb4OFY/pdb] (SYG-1 and SYG-2 complex). Unprocessed confocal or electron microscopy images are available upon request. All remaining data are included within the manuscript.

Research involving human participants, their data, or biological material

Policy information about studies with human participants or human data. See also policy information about sex, gender (identity/presentation), and sexual orientation and race, ethnicity and racism.

Reporting on sex and gender	*Use the terms sex (biological attribute) and gender (shaped by social and cultural circumstances) carefully in order to avoid confusing both terms. Indicate if findings apply to only one sex or gender; describe whether sex and gender were considered in study design; whether sex and/or gender was determined based on self-reporting or assigned and methods used.*<br>*Provide in the source data disaggregated sex and gender data, where this information has been collected, and if consent has been obtained for sharing of individual-level data; provide overall numbers in this Reporting Summary. Please state if this information has not been collected.*<br>*Report sex- and gender-based analyses where performed, justify reasons for lack of sex- and gender-based analysis.*
Reporting on race, ethnicity, or other socially relevant groupings	*Please specify the socially constructed or socially relevant categorization variable(s) used in your manuscript and explain why they were used. Please note that such variables should not be used as proxies for other socially constructed/relevant variables (for example, race or ethnicity should not be used as a proxy for socioeconomic status).*<br>*Provide clear definitions of the relevant terms used, how they were provided (by the participants/respondents, the researchers, or third parties), and the method(s) used to classify people into the different categories (e.g. self-report, census or administrative data, social media data, etc.)*<br>*Please provide details about how you controlled for confounding variables in your analyses.*
Population characteristics	*Describe the covariate-relevant population characteristics of the human research participants (e.g. age, genotypic information, past and current diagnosis and treatment categories). If you filled out the behavioural & social sciences study design questions and have nothing to add here, write "See above."*
Recruitment	*Describe how participants were recruited. Outline any potential self-selection bias or other biases that may be present and how these are likely to impact results.*
Ethics oversight	*Identify the organization(s) that approved the study protocol.*

Note that full information on the approval of the study protocol must also be provided in the manuscript.

Field-specific reporting

Please select the one below that is the best fit for your research. If you are not sure, read the appropriate sections before making your selection.

☒ Life sciences ☐ Behavioural & social sciences ☐ Ecological, evolutionary & environmental sciences

For a reference copy of the document with all sections, see nature.com/documents/nr-reporting-summary-flat.pdf

Life sciences study design

All studies must disclose on these points even when the disclosure is negative.

Sample size	For cryo-ET:<br>For wild-type nephrocytes, 14 lamellae were produced out of 10 garland nephrocyte chains. Data was acquired on 9 of the 14 lamellae (from 6 larvae).<br>For Dot;Gal80ts>sns-RNAi nephrocytes, 5 lamellae were produced out of 3 garland nephrocyte chains. Data was acquired on 2 of the 5 lamellae (from 2 larvae).<br>For pros>sns-RNAi nephrocytes, 5 lamellae were produced out of 4 garland nephrocyte chains. Data was acquired on 4 of the 5 lamellae (from 3 larvae).<br>For Rab5-RNAi nephrocytes, 3 lamellae were produced out of 2 garland nephrocyte chains. Data was acquired on 2 of the 3 lamellae (from 2 larvae).<br><br>For light microscopy images, for each genotype 6-8 animals were dissected at one time and subsequently stained and imaged. This setup was successfully repeated 3 times for each genotype or specific staining.
Data exclusions	For cryo-ET data analysis, classification was used to segregate the data set.<br>For light microscopy data, a representative image for the individual data set/ genotype was chosen. No data was per se excluded.
Replication	For cryo-ET analysis of the slit diaphragm in wild-type nephrocytes, 45 tilt-series were recorded, and 16 cryo-electron tomograms were used for the final analysis. From those, 848 sub-tomograms were extracted for averaging with RELION-5, out of which the best 595 subtomograms were selected by classification.<br>For cryo-ET analysis of Dot;Gal80ts>sns-RNAi nephrocytes, 6 tilt series displaying SDs were acquired, and images of 1 tomographic reconstruction is shown.<br>For cryo-ET analysis of pros>sns-RNAi nephrocytes, 24 tilt series were acquired, and images of 2 tomographic reconstructions are shown.<br>For cryo-ET analysis of Rab5-RNAi nephrocytes, 16 tilt series were acquired, and images of 2 tomographic reconstructions are shown.<br><br>For each genotype in the light microscopy images, 6-8 animals were used for one set. Each set was repeated at least 3 times.
Randomization	Averages were done with random individual half sets.
Blinding	For cryo-ET data analysis and the selection of subtomograms investigators were not blinded. Analysis was performed computationally.<br>For light microscopy images, no blinding was performed as there was no statistical analysis performed.

Reporting for specific materials, systems and methods

We require information from authors about some types of materials, experimental systems and methods used in many studies. Here, indicate whether each material, system or method listed is relevant to your study. If you are not sure if a list item applies to your research, read the appropriate section before selecting a response.

Materials & experimental systems

n/a	Involved in the study	
☐	☒	Antibodies
☒	☐	Eukaryotic cell lines
☒	☐	Palaeontology and archaeology
☐	☒	Animals and other organisms
☒	☐	Clinical data
☒	☐	Dual use research of concern
☒	☐	Plants

Methods

n/a	Involved in the study	
☒	☐	ChIP-seq
☒	☐	Flow cytometry
☒	☐	MRI-based neuroimaging

Antibodies

Antibodies used	mouse anti-Myc (Cell Signaling Technologies #2276), rabbit anti-Kirre (Wolff et al, 2025; PMID: 40316169), guinea pig anti-Sns (Milosavljevic et. al, 2022; PMID: 36137753)
Validation	Validation was done using immunofluorescence employing genetic backgrounds for negative and positive controls.

Animals and other research organisms

Policy information about studies involving animals; ARRIVE guidelines recommended for reporting animal research, and Sex and Gender in Research

Laboratory animals	Drosophila melanogaster L3 stadium larvae [Fly strains: tubP-GAL80ts (Bloomington Drosophila Stock Center #7019), UAS-EGFP-RNAi (Bloomington Drosophila Stock Center #41553), Dorothy-GAL4 (Bloomington Drosophila Stock Center #6903), UAS-Rab5-

RNAi(Bloomington Drosophila Stock Center #34832), Myc-Sns(Lang et al., 2022; PMID: 35876643), UAS-sns-RNAi (Vienna Drosophila RNAi Center #109442), UAS-sns-RNAi (Bloomington Drosophila Stock Center #64872), Prospero-GAL4(Weavers et. al., 2009; PMID:189719291)]

Wild animals	The study did not involve wild animals.
Reporting on sex	Sex was not considered in this study.
Field-collected samples	The study did not involve samples collected from the fields.
Ethics oversight	Experimental studies in Drosophila melanogaster are not subject to regulatory oversight and do not require ethics approval.

Note that full information on the approval of the study protocol must also be provided in the manuscript.

Plants

Seed stocks	*Report on the source of all seed stocks or other plant material used. If applicable, state the seed stock centre and catalogue number. If plant specimens were collected from the field, describe the collection location, date and sampling procedures.*
Novel plant genotypes	*Describe the methods by which all novel plant genotypes were produced. This includes those generated by transgenic approaches, gene editing, chemical/radiation-based mutagenesis and hybridization. For transgenic lines, describe the transformation method, the number of independent lines analyzed and the generation upon which experiments were performed. For gene-edited lines, describe the editor used, the endogenous sequence targeted for editing, the targeting guide RNA sequence (if applicable) and how the editor was applied.*
Authentication	*Describe any authentication procedures for each seed stock used or novel genotype generated. Describe any experiments used to assess the effect of a mutation and, where applicable, how potential secondary effects (e.g. second site T-DNA insertions, mosiacism, off-target gene editing) were examined.*
